# Therapeutic strategies for glucose transporter 1 deficiency syndrome

**DOI:** 10.1002/acn3.50881

**Published:** 2019-08-28

**Authors:** Maoxue Tang, Sarah H. Park, Darryl C. De Vivo, Umrao R. Monani

**Affiliations:** ^1^ Department of Pathology & Cell Biology Columbia University Medical Center New York New York 10032; ^2^ Center for Motor Neuron Biology and Disease Columbia University Medical Center New York New York 10032; ^3^ Department of Neurology Columbia University Medical Center New York New York 10032

## Abstract

Proper development and function of the mammalian brain is critically dependent on a steady supply of its chief energy source, glucose. Such supply is mediated by the glucose transporter 1 (Glut1) protein. Paucity of the protein stemming from mutations in the associated *SLC2A1* gene deprives the brain of glucose and triggers the infantile‐onset neurodevelopmental disorder, Glut1 deficiency syndrome (Glut1 DS). Considering the monogenic nature of Glut1 DS, the disease is relatively straightforward to model and thus study. Accordingly, Glut1 DS serves as a convenient paradigm to investigate the more general cellular and molecular consequences of brain energy failure. Here, we review how Glut1 DS models have informed the biology of a prototypical brain energy failure syndrome, how these models are facilitating the development of promising new treatments for the human disease, and how important insights might emerge from the study of Glut1 DS to illuminate the myriad conditions involving the Glut1 protein.

## The Genetics and Clinical Phenotype of Glut1 DS

The mammalian brain relies mainly on glucose to fuel its energy requirements.^1^ An adequate supply of this energy source is particularly important during infancy and early childhood.^2^ This period of postnatal development witnesses a profound increase in the size and complexity of the brain as new synaptic connections are made and the overall cerebral circuitry is sculpted and refined. A paucity of brain glucose (neuroglycopenia) during these early postnatal years starves the organ of energy and likely arrests the development of neuronal circuits underlying the diverse repertoire of coordinated movements and complex behaviors that distinguishes humans from other species. One way to begin to understand the cellular pathology and molecular mechanisms associated with neuroglycopenia is through the study of monogenic causes of the condition. Glucose transporter 1 deficiency syndrome (Glut1 DS) is the quintessential example. Striking primarily in infancy or childhood, Glut1 DS, also known as De Vivo Syndrome, was initially described in the early 1990s to comprise epileptiform seizures, developmental delay, and a complex movement disorder that combined elements of spasticity, ataxia, and dystonia.^3^ All Glut1 DS patients are found to exhibit hypoglycorrhachia – reduced (<60 mg/dL or 3.3 mmol/L; ~90% have <40 mg/dL or 2.2 mmol/L) levels of glucose in the cerebrospinal fluid (CSF). Lactate levels in CSF are generally reported to be in the low to low‐normal range (<9 mg/dL or 0.5 mmol/L).^4^

In 1998, haploinsufficiency of the *SLC2A1* gene and thus low levels of its translated product, the Glut1 protein, were found to underlie Glut1 DS.^5^ While most patients harbor de novo mutations of the gene, they may also inherit the disease in an autosomal dominant manner.^6^ In rare instances an autosomal recessive pattern of Glut1 DS inheritance is observed and may result in compound heterozygotes.^7^, ^8^ Such compound heterozygotes, nevertheless, exhibit residual Glut1 activity; complete absence of the protein has never been reported, and consistent with its widespread expression and housekeeping function, is embryonically lethal.^9^, ^10^ The identification of the genetic cause of Glut1 DS has facilitated accurate diagnosis of the disease. It has also resulted in the recognition of a greatly expanded Glut1 DS clinical phenotype.^11^ Indeed, it is now clear that there is a spectrum of disease phenotypes ranging from those observed in the classic form of Glut1 DS to features such as nonepileptic, paroxysmal exercise‐induced dyskinesias, hereditary spastic paraplegia, and hemolytic anemias.^12^, ^13^, ^14^ Appreciation of these newer phenotypes combined with recent reports that *SLC2A1* mutations associate with ~10% of absence epilepsies^15^ and about 1% of idiopathic generalized epilepsies (IGE)^16^ suggest that Glut1 DS may be significantly more prevalent than previously thought. Assuming a lifetime epilepsy prevalence of ~7 per 1000 individuals^17^ and estimates that 15–20% of these constitute IGEs,^18^ the Glut1 DS patient population in the US may range from 3400 to 4500 individuals. This estimate would be equivalent to an incidence of approximately 1 per 75,000 births. As the disease is not recognized to be anymore widespread in specific ethnic groups, it is expected that the current worldwide population of individuals afflicted with Glut1 DS is ~105,000.

While convention dictates that a genuine case of Glut1 DS stems from molecular lesions in the *SLC2A1* gene, mutations in the gene are not essential in triggering a clinical phenotype consistent with Glut1 deficiency.^19^ In certain instances, Glut1 DS‐like patients are found to express reduced Glut1 despite a normal protein coding sequence.^20^, ^21^ This is suggestive of noncoding *SLC2A1* mutations or perturbations in factors that regulate Glut1 expression. It is also conceivable that novel mechanisms, possibly acting on Glut1 activity rather than on its expression, explain the hypoglycorrhachia and clinical phenotype of Glut1 DS. Investigating the cause of the disease in such “exception” patients will likely be especially informative as researchers attempt to determine precisely how Glut1 deficiency causes selective brain dysfunction and the neurodevelopmental phenotype characteristic of Glut1 DS.

## The Pathology of Glut1 DS: Outcomes From the Study of Model Organisms

Even patients who occupy the severe end of the Glut1 DS spectrum generally have a normal lifespan. Moreover, autopsy material from patients is limited and little described. Thus, aside from brain imaging studies of these patients, information on the pathology of Glut1 DS has relied mainly on animal models of the disease. Studies that *have* imaged the human Glut1 DS brain using ^18^F‐fluorodeoxyglucose (FDG) positron emission tomography (PET) reveal a generally reduced uptake of the radiolabel in the cortex accompanied by particularly severe hypometabolism of the mesial temporal regions, cerebellum, and thalamus. This contrasts with apparent signal hyperintensity in the basal ganglia.^22^, ^23^ The study further demonstrated that the distinctive metabolic footprint of Glut1 DS appears in infancy and is essentially immutable. Magnetic resonance imaging of the brains of the patients did not uncover significant abnormalities despite the microcephaly that was observed. This suggests the development of an otherwise normal gross cerebral structure.

**Figure 1:**
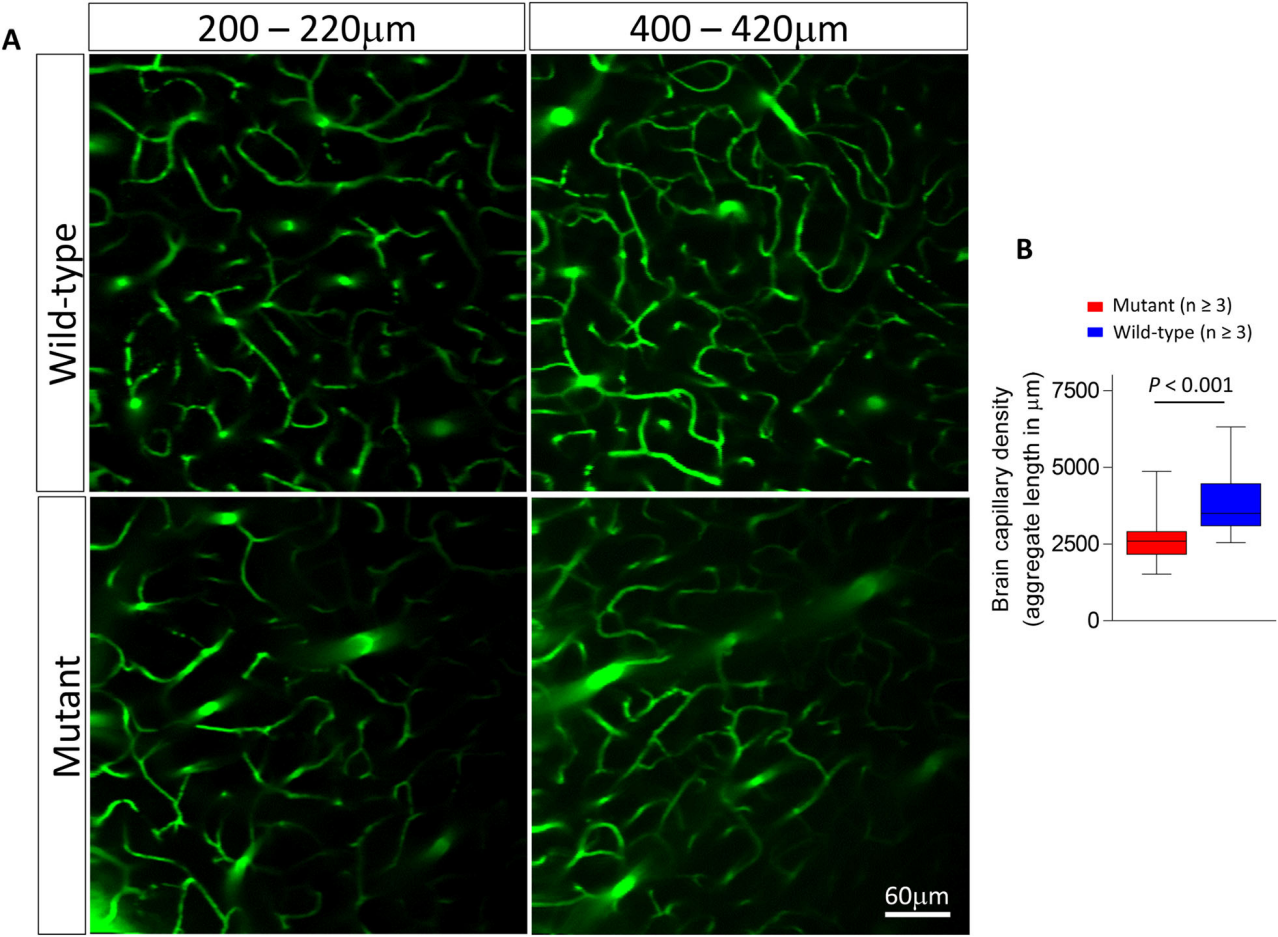
A smaller brain microvasculature resulting from Glut1 paucity. (A) two‐photon images of cortical brain capillaries visualized following an injection of FITC–dextran into the circulation of live wild‐type or Glut1 DS model mice. Vessels were imaged at the indicated depths from the surface of the cortex. (B) Quantification of cortical brain capillary density in wild‐type and Glut1 DS model mice illustrates the smaller cerebral microvasculature in mutants haploinsufficient for the *Slc2a1* (Glut1) gene. Glut1 DS, glucose transporter 1 deficiency syndrome.

While studies on Glut1 DS model mice have largely confirmed the poor glucose uptake seen in the patient brain, these studies also suggest an intriguing cerebral pathology. Perhaps the most striking finding involves the brain microvasculature.^24^, ^25^ Haploinsufficiency of Glut1 arrests brain angiogenesis resulting in a relatively diminutive cerebral microvasculature (Fig. 1). However, considering (1) the particularly abundant expression of Glut1 in brain endothelial cells,^26^ (2) the importance of a subset of these cells – endothelial tip cells – in expansion of the microvasculature, and (3) the near‐absolute reliance of tip cells on Glut1 to pioneer new blood vessels,^27^ the consequences of Glut1 paucity on the cerebral microvasculature is perhaps not altogether surprising. Whether low Glut1 in endothelial cells alone is sufficient to trigger this pathology remains to be investigated, as the protein is reported to be expressed, albeit at low levels, in additional brain cells including oligodendrocytes, microglia, and ependymal cells.^28^, ^29^, ^30^ Still, it is clear that a ~50% loss of Glut1 does not impair the blood–brain barrier (BBB).^24^ In contrast, severe (>90% knockdown) of Glut1, as effected in a zebrafish model, not only resulted in loss of cerebral endothelial cells, downregulation of tight‐junction proteins that ensure an intact BBB and thus vasogenic edema, but also a disruption of the ocular vasculature.^31^, ^32^ However, such profound loss of Glut1 is not a characteristic of the human disease, and the pathology observed in the fish model, while instructive, is unlikely to be reflective of human Glut1 DS.

**Figure 2:**
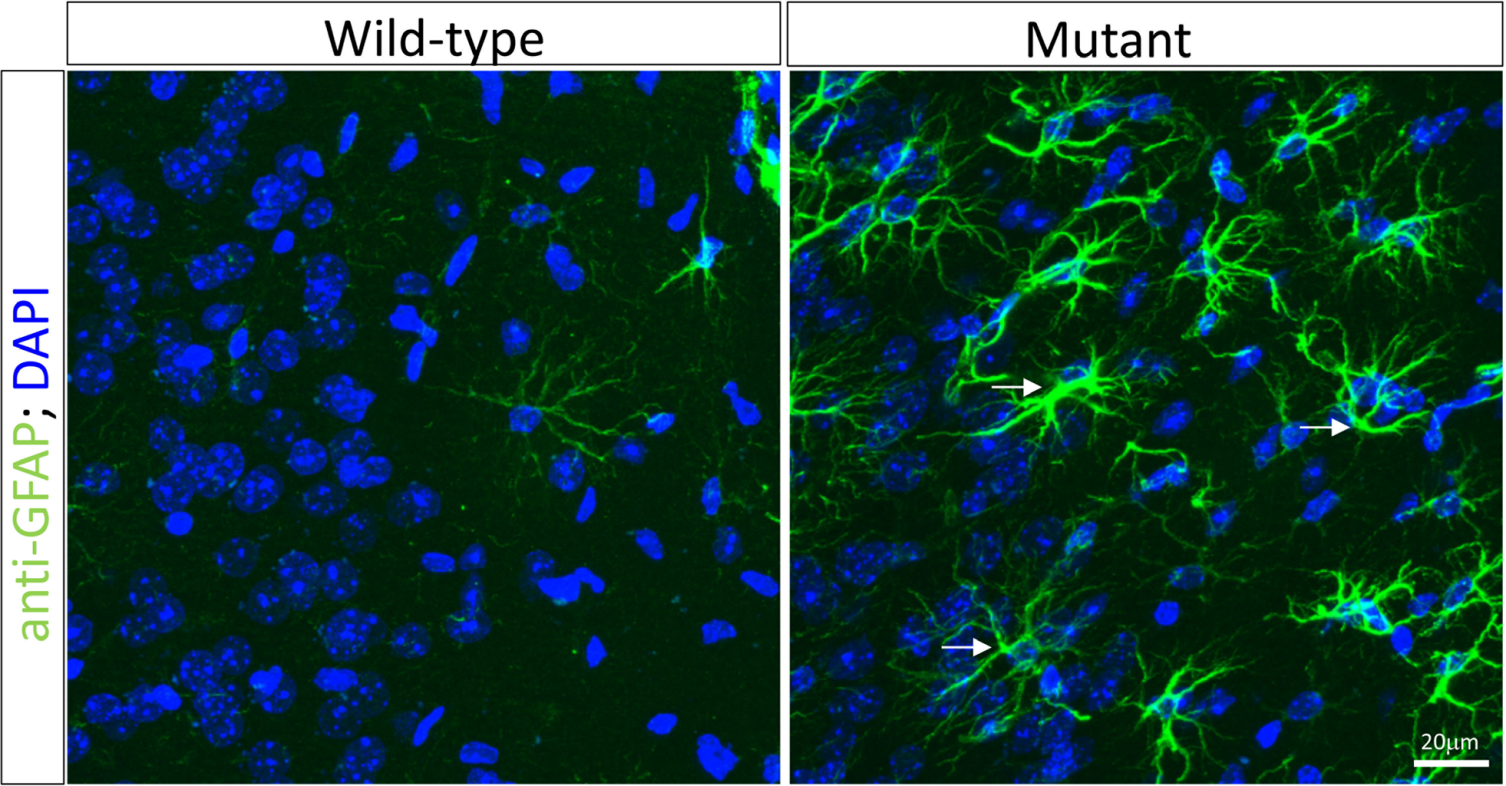
Gliosis resulting from Glut1 deficiency. Confocal images of the cortical brain from an approximately 10‐month‐old Glut1 DS model mouse and a littermate control depict evidence of neuroinflammation in the mutant. Observed are markedly greater numbers of reactive astrocytes (arrows) in the Glut1 DS brain. Glut1 DS, glucose transporter 1 deficiency syndrome.

A combination of reduced (~50%) Glut1 in cerebral endothelial cells and fewer brain capillaries would be expected to deprive the brain of the energy resources required in properly establishing its circuitry. Although it is unclear precisely how this occurs, the Glut1 DS brain is observed to be significantly reduced in size.^9^ The cause of such micrencephaly is reported to involve cell loss, but this finding is debated.^25^, ^33^ In contrast, evidence of gliosis in the Glut1 brain is clear^33^ (Fig. 2). However, whether the neuroinflammation is a primary consequence of reduced Glut1 or a relatively downstream and nonspecific event, must be empirically determined. It will also be critically important to eventually examine patient brains to determine if gliosis and a diminutive microvasculature are genuine pathological features of human Glut1 DS, and thus aspects of the disease amenable to therapeutic intervention. In the absence of human autopsy material to investigate such pathology, imaging techniques may prove instructive. Thus, for instance, the microvasculature could potentially be examined using arterial spin labeling to measure cerebral perfusion, while the extent of neuroinflammation may be gleaned by exploiting PET imaging and ligands such as ^11^C‐PK11195 and ^11^C‐deuterium‐l‐deprenyl which bind the microglial translocator protein and astrocytic monoamine oxidase B proteins respectively.^34^ We are exploring each of these in model mice as well as in the human patient in present work.

## Current and Emerging Therapies for Glut1 DS

### Ketogenic diet

The current standard of care for Glut1 DS is the ketogenic diet, a high‐fat diet that raises levels of ketone bodies in the blood to make them available to the brain.^35^, ^36^ Ketones such as β‐hydroxybutyrate and acetoacetate are alternate, albeit imperfect, substitutes for brain glucose. Ketones traverse the BBB through a different transporter, the monocarboxylate transporter 1 (MCT1) and fuel the brain by serving as a source of acetyl CoA which is eventually fed into the tricarboxylic acid (TCA) cycle. When administered early during the course of the disease, the ketogenic diet attenuates the seizures associated with Glut1 DS. However, its effects on other disease characteristics are variable and modest.^37^ There are several possible reasons for this. The first stems from a continued paucity of glycolytic intermediates which derive exclusively from glucose and potentially underlie specific disease characteristics. This is consistent with the dependence of endothelial tip cells on glycolysis as a source of energy. A second is that glucose itself and/or Glut1 serve as signaling molecules vital for the well‐being of the developing brain. A final explanation lies in the rapid downregulation of the MCT1 protein once the organism is weaned.^38^ This would eventually preclude the entry of adequate ketone bodies into the cerebral parenchyma, exacerbating the disease condition imposed on the brain by Glut1 paucity. Additionally, long‐term treatment with the ketogenic diet is not only challenging owing to noncompliance but also may be associated with adverse consequences including significant reduction of bone mass, cardiovascular complications due to atherosclerosis, and in rare instances, the induction of a state of coma.^39^, ^40^, ^41^ Thus it is clear that notwithstanding the widespread use of the diet in treating Glut1 DS, there is a clear unmet need for other therapies for the disease.

### Triheptanoin

Triheptanoin is an odd‐chain (C7) triglyceride that serves as an anaplerotic agent to replenish the metabolic intermediates of the TCA cycle.^42^ Promoted as an alternative to the ketogenic diet because it can be metabolized into both acetyl CoA as well as propionyl CoA – a source of C3 ketone bodies that easily traverse the BBB through MCT1 – triheptanoin, in an open‐label study, did reduce spike wave discharges in patients.^43^ However, a randomized, blinded, placebo controlled clinical trial to assess the effects of the drug on Glut1 DS patients experiencing seizures or disabling paroxysmal movement disorders failed to demonstrate the benefit suggesting that triheptanoin is of limited therapeutic use.^44^

### Gene and protein replacement

**Figure 3:**
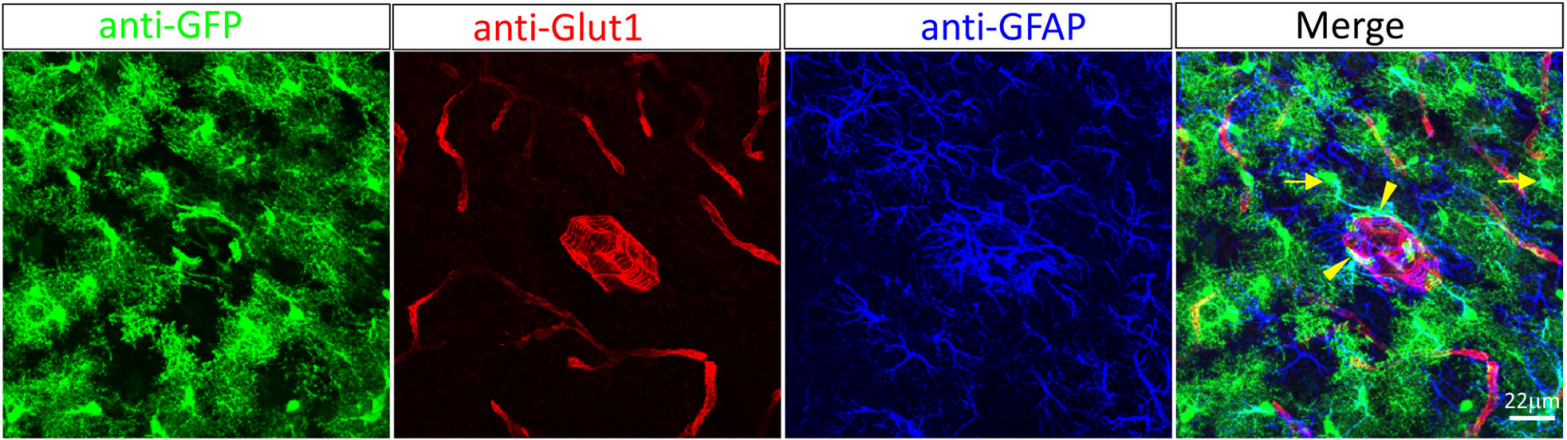
AAV9‐mediated transduction of brain cells. An AAV9‐eGFP construct systemically administered into a PND3 mouse transduces astrocytes (arrows) and brain capillaries (arrowheads). AAV9, adeno‐associated virus serotype 9.

Considering the cause of Glut1 DS–Glut1 paucity, restoring the protein by means of gene replacement is intuitively appealing. Moreover, the advent of viral vectors, for example, adeno‐associated virus serotype 9 (AAV9), which efficiently transduce a variety of cell types (Fig. 3 and Refs. 45, 46) and serve as convenient vehicles for delivering human genes,^47^ provide a fillip for gene replacement as a means to treat Glut1 DS. Indeed, proof‐of‐concept studies by us and others have demonstrated the promise of gene replacement for the disease.^24^, ^48^ We found that administering AAV9‐Glut1 to young postnatal model mice raised cerebral Glut1 expression, increased concentrations of CSF glucose, facilitated brain growth, enhanced uptake of glucose into the mutant brain, and erased evidence of motor abnormalities. Repletion of the protein also restored the brain microvasculature to its normal size and complexity.^24^ Translating these findings into a treatment for the human disease is a logical extension of the preclinical work and is currently being pursued by us and others.

Viral vector‐mediated gene replacement is a clinically proven means of treating diseases caused by protein paucity.^47^ Direct replenishment of the relevant protein could, in principle, also constitute a treatment. Thus, for example, Glut1 could be delivered to cells by fusing it to a cell‐penetrating peptide such as Tat, the protein transduction domain of the human immunodeficiency virus.^49^ Preclinical work on model mice could prove instructive in this regard.

### Small molecules and biologics to enhance Glut1 expression

Amongst the small molecules used in the treatment of Glut1 DS is acetazolamide, a carbonic anhydrase inhibitor and anticonvulsant that promotes ion transport across the BBB thus altering intracellular pH. Although it is unclear precisely how acetazolamide mitigates disease in Glut1 DS, it was used to successfully attenuate paroxysmal dyskinesia in a patient with the disease.^50^

Small molecules may also be used to increase Glut1 expression and/or its activity. This is especially feasible from a therapeutics perspective considering the presence of at least one intact *SLC2A1* gene in most Glut1 DS patients, and could be informed by the abundant literature describing molecular pathways that converge onto the Glut1 protein. One small molecule that has been recommended for the treatment of the disease based on its ability to stimulate Glut1/4 translocation and thus enhance glucose uptake is alpha lipoic acid (thioctic acid).^6^, ^51^ Glut1 translocation to the membrane of brain endothelial cells, likely key to its ability to facilitate glucose transport, is also effected by the insulin‐like growth factor 1 (IGF‐1R). IGF‐1R knockdown enhances cell surface Glut1 levels, an effect possibly mediated by GAIP‐interacting protein C‐terminus (GIPC), a protein known to bind both the PDZ domain of Glut1 and IGF‐1R.^52^ Loss of the PDZ domain or deficiency of GIPC decreased cell surface Glut1 levels and resulted in targeting of the protein to lysosomes.^53^ Trafficking of cytoplasmic Glut1 stores to the membrane is also mediated by protein kinase C‐dependent phosphorylation of S226 of the transporter on the one hand and by autophagy on the other.^54^, ^55^ In the latter instance, LC3+ autophagic compartments bind and sequester the RabGAP protein TBC1D5, thus disrupting the inhibitory interaction between the latter and the retromer complex and enabling retromer‐bound Glut1 to be recycled to the cell surface.

An increase in absolute Glut1 levels, including the membrane‐bound pool, reliably occurs under hypoxia and expression of the hypoxia‐inducible factor 1α (Hif1α) transcription factor.^56^ Hif1α is reported to be the final mediator of a number of signaling pathways regulating Glut1 expression. These commonly involve GSK‐3, the tuberous sclerosis complex (Tsc1/2) and mTORC1.^57^ Inhibitors of GSK3β result in decreased phosphorylation of Tsc2, enhanced mTOR signaling, an increase in Hif1α, and ultimately, increased Glut1 expression. A parallel cascade that is initiated by BMP2 signaling and involves Smad4, Pten, mTORC1, and Hif1α also raises Glut1 levels and was shown to be essential for skeletal muscle development.^58^ Finally, a number of reports have demonstrated the effects of FGF21 on the transcriptional activation of Glut1. FGF21 acts, at least in part, by inducing the phosphorylation of the transcription factors SRF and Elk‐1 which bind elements in the Glut1 promoter to activate transcription.^59^ FGF21 mimetics could, in principle, evoke the same effects. A number of these have been developed for obesity, dyslipidemias, and diabetes, and may find use for Glut1 DS too.^60^ Similarly, small molecules that alter one or more of the signaling pathways identified here could become a means of enhancing Glut1 expression for the treatment of haploinsufficient Glut1 DS. Considering the ~12% of total Glut1 that is cytoplasmic and can presumably be mobilized to the endothelial cell surface to effect glucose transport,^61^ and the nominal (~25%) increase in Glut1 expression required to erase disease symptoms,^19^ a small molecule or biologic enhancer of Glut1 expression/activity need only have a modest effect to be of significant therapeutic value. We advocate a careful testing of existing molecules as well as de novo screens for new ones to identify compounds that increase Glut1 expression for the treatment of Glut1 DS. While agents that emerge from these screens will almost certainly include those which directly act on the Glut1 gene and/or protein, a number may also increase Glut1 indirectly by acting on interactors of the transporter. A great many such interactors have been identified^62^ and listed in public databases, for example, PubMed. Some such as TXNIP, which binds Glut1 and suppresses its activity by inducing internalization through clathrin‐coated pits, have known mechanisms of action.^63^ However, in most instances, the interactors have merely been revealed through mass spectrometry or yeast two‐hybrid experiments. The physiological relevance of these interactions remains to be empirically determined.

## The Case for Defining the Temporal and Spatial Requirements for Glut1 in Therapy Development

To maximize the therapeutic effects of an enhancer of Glut1 activity and or function, one must ensure that treatment of Glut1 DS with the agent is initiated in a timely manner, sustained appropriately, and delivered to relevant tissues. The rationale for doing so is amply demonstrated in effectively treating another neurological disease, spinal muscular atrophy, which is also caused by paucity of a housekeeping protein.^64^ Accordingly, it is not only prudent but also critical to precisely define the spatial and temporal requirements for the Glut1 protein. While these will only become truly apparent in the human patient, much can be gleaned from studies in model mice. One important AAV9‐based study has already demonstrated the early requirements for Glut1 to thwart disease.^24^ Early, postnatal AAV9‐mediated Glut1 restoration was most effective. Replenishing protein during adulthood was essentially without therapeutic effect despite restoring levels of CSF glucose. Similar studies involving repletion and/or depletion of the Glut1 protein will be required to define the critical cellular sites of action of the protein. Determining the contributing effects of two cell types – brain endothelia and astrocytes – based on the abundant expression of Glut1 in these cells and their role in supplying energy to brain neurons will be particularly important. These studies will likely also be instructive in determining to what extent Glut1 levels can be safely raised to avoid any unintended effects of overexpressing the protein, as glucose transporters are known to be upregulated in cancers.^65^

## Altered Glut1 Biology: Relevance Beyond Glut1 DS

Although Glut1 DS is perhaps the most direct consequence of altered (reduced) Glut1 protein, the biology and regulation of the protein assumes relevance well beyond the neurodevelopmental disorder. Indeed, perturbations in Glut1 levels have been implicated in conditions as diverse as diabetes, cancer, retinitis pigmentosa (RP), and Alzheimer's disease (AD). Thus, for instance, tumors of diverse origins are widely reported to upregulate Glut1 to promote cellular proliferation, and increased expression of the transporter often correlates with poor prognosis.^65^ and refs therein Targeted knockdown of Glut1 has, therefore, been proposed as one way of arresting tumor growth.^66^ In contrast, in diabetes and many forms of cardiac disease, glucose transporters, including Glut1, are decreased in expression – especially in the myocardium.^67^ However, it is in the context of two other conditions, one relatively rare (RP), the other increasingly common in aging populations (AD) that preclinical work on Glut1 DS may assume particular relevance.

Retinitis pigmentosa is a group of rare (~1:4000) genetic disorders that involve degeneration and loss of rods and cones, the photoreceptors of the eye. Mutations in multiple genes can cause the condition. However, a common pathway to retinal degeneration in all forms of RP may involve a rod‐derived cone viability factor (RdCVF). Acting through its receptor, Basigin‐1, which in turn binds cell‐surface Glut1, RdCVF serves as a trophic factor for the cones of the eye.^68^ These photoreceptors are highly glycolytic and thus extremely sensitive to glucose deprivation. Small molecules or biologics engineered to raise Glut1 levels for Glut1 DS could, therefore, very likely find additional use for RP.

Alzheimer's disease is a second neurological disease reported to involve perturbed (decreased) expression of Glut1. Currently, patients with AD have little recourse; AD is the one disease amongst the top 10 causes of death in humans for which there is neither a prevention nor effective treatment.^69^ Among the many explanations for the underlying cause of AD is the neuroenergetic hypothesis – that decreased availability of usable energy for the brain ultimately triggers the chain of events that lead to the disease.^70^ In this vein, it is instructive to note that despite constituting just 2% of the overall mass of the human body, the brain consumes some 25% of the energy utilized by a healthy individual.^1^ It is also worth remembering that the brain mostly uses glucose and is thus heavily reliant on an intact cerebral microvasculature and on Glut1 to ensure a steady supply of the nutrient. Accordingly, it is interesting to note that the AD brain is not only widely reported to be deficient in Glut1 and hypometabolic – in ^18^F‐FDG PET imaging studies^71^, ^72^, ^73^, ^74^, ^75^ – but also appears hypoperfused.^76^, ^77^, ^78^, ^79^, ^80^, ^81^ Moreover, at least one study found an inverse correlation between CSF levels of the AD‐causing Aβ peptide and CSF glucose, suggestive of low concentrations of glucose in the CSF of patients with AD. Low CSF glucose is, of course, a signature feature of Glut1 DS, and similar findings in AD patients indicate at least some shared mechanisms between the two diseases. The precise sequence of pathological events in the brain that eventually lead to a diagnosis of AD remain to be determined. However, a reduced supply of glucose is consistent with the neuroenergetic hypothesis as a means of explaining AD pathogenesis. The combination of reduced glucose and a shrinking cerebral microvasculature may also explain amyloid deposition and the tau‐containing neurofibrillary tangles that serve as hallmarks of the AD brain.^82^ The cascade of events likely spirals into a vicious cycle, with amyloid deposition further damaging brain endothelia, exacerbating Glut1 deficiency, and damaging the brain capillary network.^83^ Arresting or reversing such pathology might be effected by augmenting Glut1. Developing a means of doing so for Glut1 DS could, therefore, become useful in the context of more prominent conditions such as age‐associated dementia and AD. Considering the observations we make here, one intriguing possibility is a higher than expected incidence of dementia and/or AD amongst Glut1 DS patients. However, given early‐onset cognitive dysfunction in these patients, using cognitive ability as an outcome measure is unlikely to prove useful. Rather, future correlations may have to rely on examining the cellular structure of the Glut1 DS brain for evidence of AD‐like pathology. Still, it is these potential connections that make studying the biology and treatment of Glut1 DS relevant beyond just the one neurodevelopmental disorder.

## Author Contributions

M.T. and U.R.M. conceived of the layout and organization of the draft. U.R.M. wrote the initial draft. All authors read, amended, and approved the final manuscript.

## Conflicts of Interest

M. T. and S. H. P. have nothing to disclose. U. R. M. and D. C. D are listed as inventors on a provisional patent that describes the use of viral vector technology to treat Glut1 DS.
